# Supplementary material for: Lateral dispersion is required for circuit integration of newly generated dentate granule cells
Source: Nat Commun. 2019 Jul 25;10:3324. doi: 10.1038/s41467-019-11206-9 (PMC6658520; doi:10.1038/s41467-019-11206-9)
Supplement: Supplementary file 1 — Supplementary Information [file 41467_2019_11206_MOESM1_ESM.pdf]

Supplementary Information

Lateral dispersion is required for circuit integration of newly generated dentate granule cells

Wang et al.

Supplementary Figures

Supplementary Figure 1

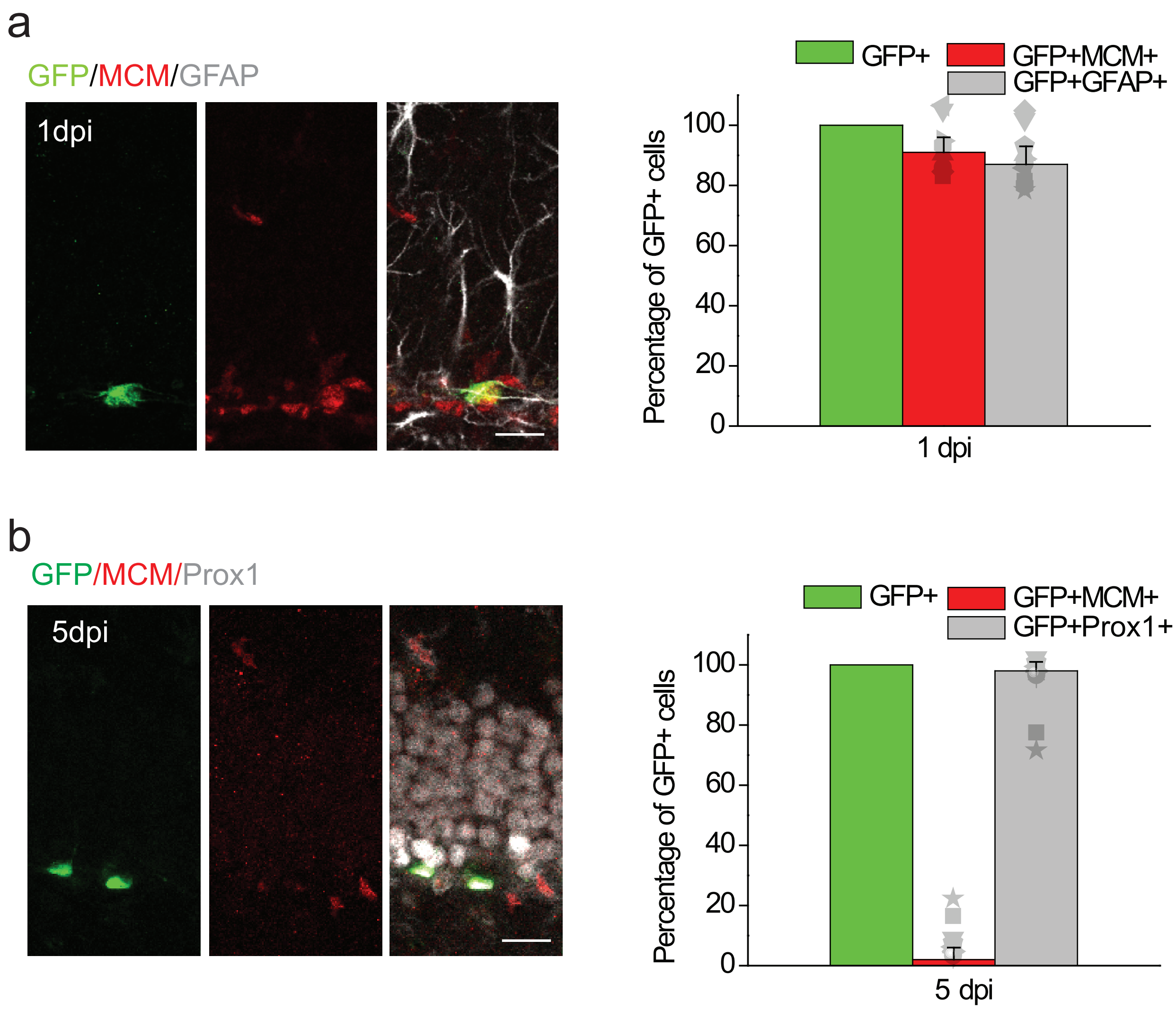

Supplementary Figure 1. Retrovirus-labeled cells at 5 dpi were immature DGCs
a) On the left are representative images of a newly generated DGC at 1 dpi. The cell is marked by a GFP-expressing retrovirus (left-most panel), and MCM+ (middle panel) and GFAP+ (right panel). The scale bar is 20μm. A summary plot showing the percentage of GFP+MCM+ and GFP+GFAP+ of GFP+ newborn cells at 1dpi is shown on the right.
b) On the left are representative images of newly generated DGCs at 5 dpi expressing GFP. These cells are MCM- (middle panel) and Prox1+ (right panel). The scale bar is 20 μm. On the right is a summary plot showing the percentage of GFP+MCM+ and GFP+Prox1+ of GFP+ newborn neurons at 5dpi. For both a and b, the averaged percentages were collected from 3 mice for each time-point. The number of GFP+ cells of each mouse was between 30-45.

Supplementary Figure 2

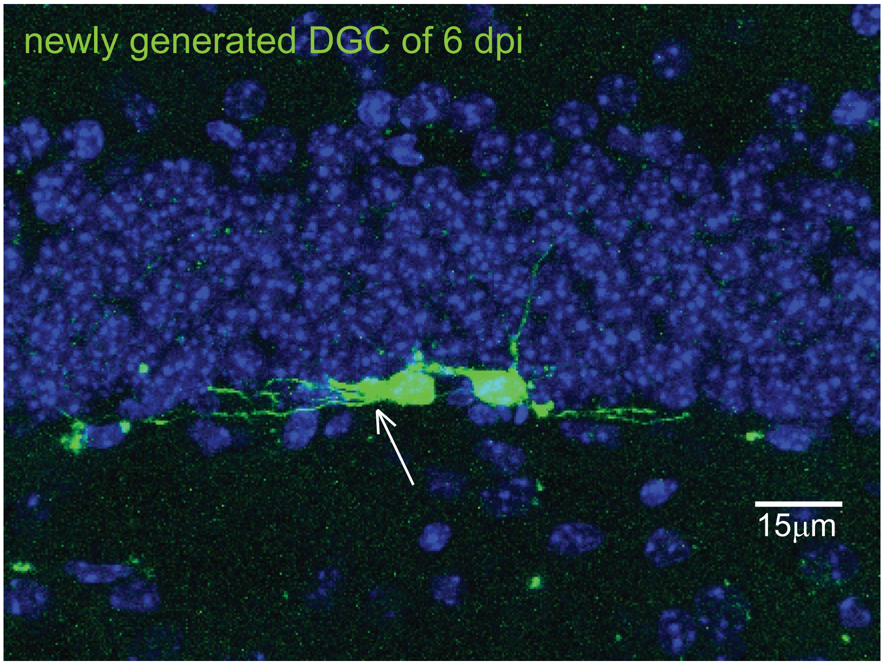

Supplementary Figure 2. Neurons showed a typical leading process with a proximal spindle expansion along the neurogenic zone

Representative image of newly generated DGCs at 6 dpi. GFP+ cells are newly generated DGCs marked by retrovirus expressing GFP. Arrow points to the expansion, which serves as the leading process for dispersion in the SGZ of the dentate gyrus.

Supplementary Figure 3

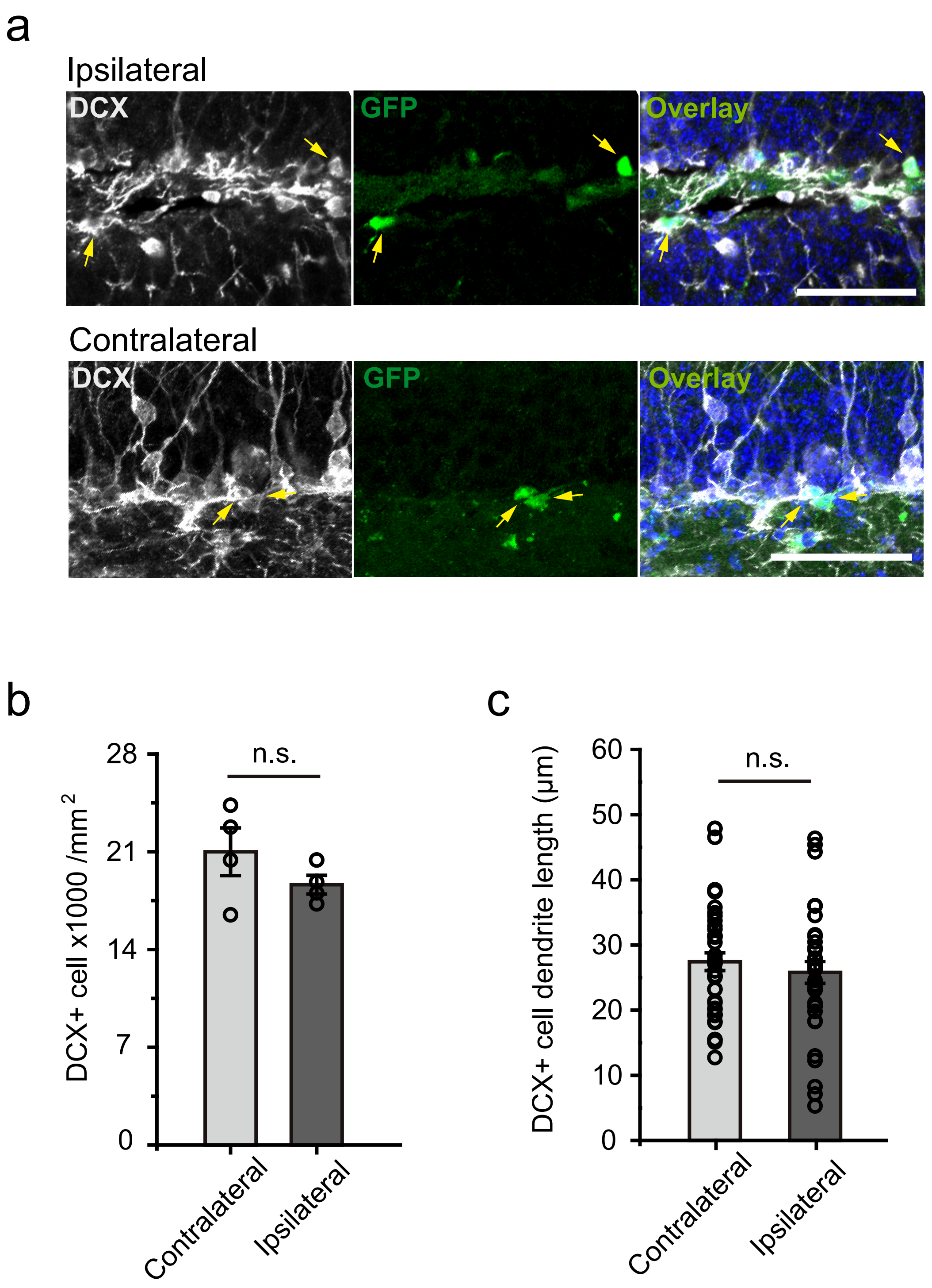

Supplementary Figure 3. DCX+ cells showed normal features after lens implantation

a) Confocal images of the dentate gyrus following GFP retrovirus injection (7 dpi) ipsilateral, and contralateral to the lens implantation site. The arrows indicate newborn DGCs expressing both GFP (Green) and DCX (Silver) with DAPI (blue) staining. The scale bar is 50 μm.

b) Quantification of DCX+ cells ipsilateral and contralateral to lens implantation (n = 4, Kruskal-Wallis test, n.s represents P > 0.05).

c) Quantification of the process length of horizontally-positioned DCX+ cells (n = 35 for ipsilateral, and 42 cells for contralateral, unpaired *t* test, n.s represents P > 0.05).

Supplementary Figure 4

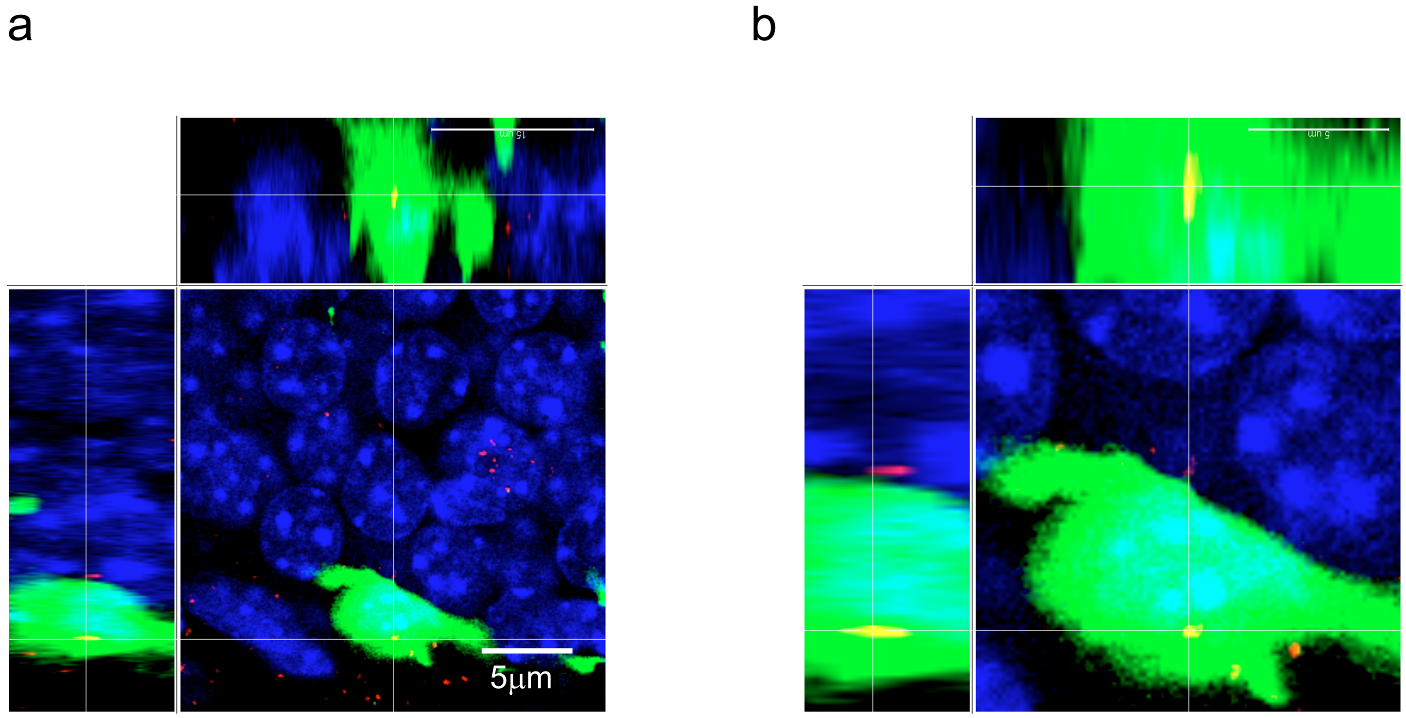

Supplementary Figure 4. Expression of CX43 in newly generated DGCs

a) Image of newly generated DGCs expressing GFP at 14 dpi. The red signal is CX43 stained with its antibody.

b) Enlarged view of the cell in panel a.

Supplementary Figure 5

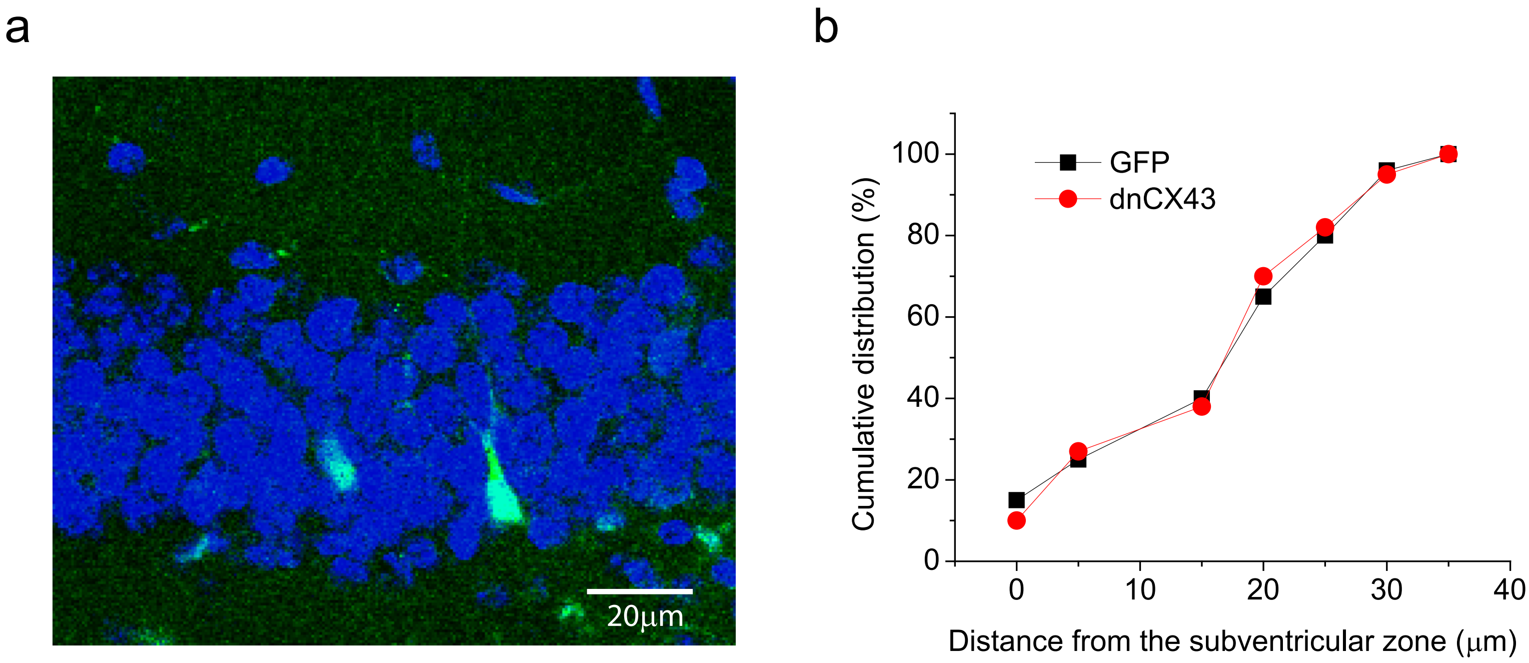

Supplementary Figure 5. Expression of dnCX43 has little effect on radial migration of newly generated DGCs

a) Representative image of newly generated DGCs expressing GFP at 14 dpi.

b) Plot of distance between the somas and the subventricular zone of newly generated DGCs expressing GFP or dnCX43 at 14 dpi. Two-tailed unpaired t test, $P > 0.05$ ($n = 3–4$).

Supplementary Figure 6

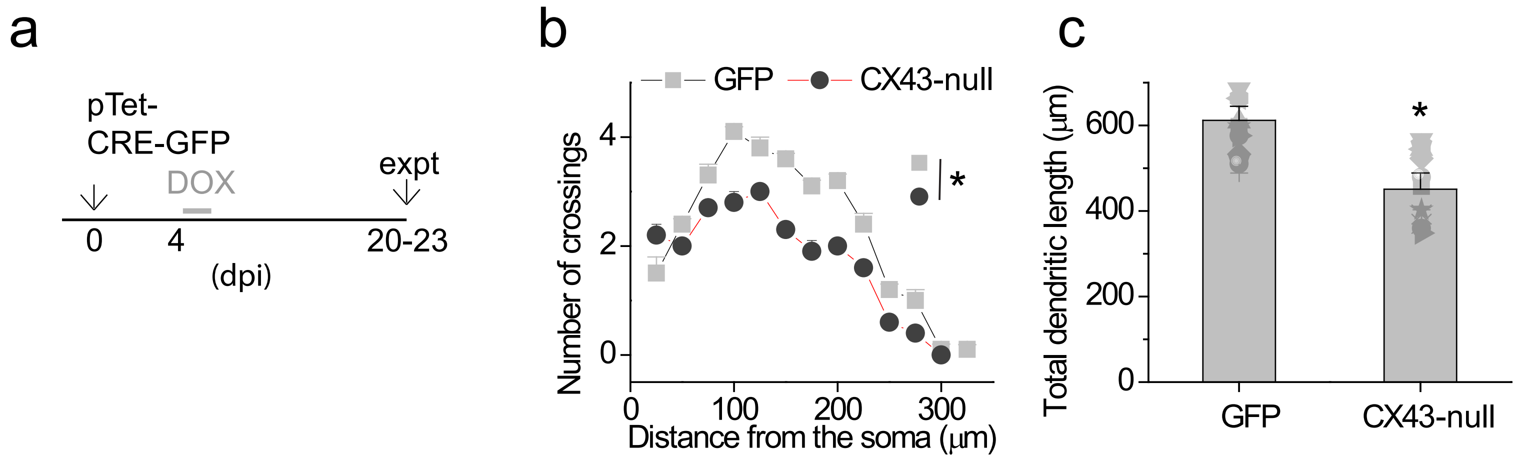

Supplementary Figure 6. Disruption of electrical coupling impairs morphological integration of newly generated DGCs

a) Experimental timeline of birth-date labeling and introduction of a Cre expression using a method with doxycycline as previously described (Kumamoto et al., 2012) in newborn DGCs of adult floxed CX43 mice.

b-c) In b, Sholl analysis of the dendritic tree of control and CX43-null at 21 dpi. In c, a summary of total dendritic length for control and CX43-null cells at 21 dpi. (n=18-25 neurons from 3 mice of each group. For b, statistical significance was determined by Student's t-test; for c, *: $p<0.01$, ANOVA). All values represent mean±SEM.
