# Supplementary material for: Lateral dispersion is required for circuit integration of newly generated dentate granule cells
Source: Nat Commun. 2019 Jul 25;10:3324. doi: 10.1038/s41467-019-11206-9 (PMC6658520; doi:10.1038/s41467-019-11206-9)
Supplement: Supplementary file 3 — Reporting Summary [file 41467_2019_11206_MOESM3_ESM.pdf]

nature research

Corresponding author(s): Shaoyu Ge

Reporting Summary

Nature Research wishes to improve the reproducibility of the work that we publish. This form provides structure for consistency and transparency in reporting. For further information on Nature Research policies, see Authors & Referees and the Editorial Policy Checklist.

Statistical parameters

When statistical analyses are reported, confirm that the following items are present in the relevant location (e.g. figure legend, table legend, main text, or Methods section).

n/a	Confirmed	
☐	☒	The exact sample size (n) for each experimental group/condition, given as a discrete number and unit of measurement
☐	☒	An indication of whether measurements were taken from distinct samples or whether the same sample was measured repeatedly
☐	☒	The statistical test(s) used AND whether they are one- or two-sided<br>*Only common tests should be described solely by name; describe more complex techniques in the Methods section.*
☐	☒	A description of all covariates tested
☐	☒	A description of any assumptions or corrections, such as tests of normality and adjustment for multiple comparisons
☐	☒	A full description of the statistics including central tendency (e.g. means) or other basic estimates (e.g. regression coefficient) AND variation (e.g. standard deviation) or associated estimates of uncertainty (e.g. confidence intervals)
☐	☒	For null hypothesis testing, the test statistic (e.g. F, t, r) with confidence intervals, effect sizes, degrees of freedom and P value noted<br>*Give P values as exact values whenever suitable.*
☒	☐	For Bayesian analysis, information on the choice of priors and Markov chain Monte Carlo settings
☒	☐	For hierarchical and complex designs, identification of the appropriate level for tests and full reporting of outcomes
☒	☐	Estimates of effect sizes (e.g. Cohen's d, Pearson's r), indicating how they were calculated
☐	☒	Clearly defined error bars<br>*State explicitly what error bars represent (e.g. SD, SE, CI)*

Our web collection on statistics for biologists may be useful.

Software and code

Policy information about availability of computer code

Data collection	No software was used.
Data analysis	No software was used.

For manuscripts utilizing custom algorithms or software that are central to the research but not yet described in published literature, software must be made available to editors/reviewers upon request. We strongly encourage code deposition in a community repository (e.g. GitHub). See the Nature Research guidelines for submitting code & software for further information.

Data

Policy information about availability of data

All manuscripts must include a data availability statement. This statement should provide the following information, where applicable:

- Accession codes, unique identifiers, or web links for publicly available datasets
- A list of figures that have associated raw data
- A description of any restrictions on data availability

The data that support the findings of this study are available from the corresponding author upon reasonable request.

Field-specific reporting

Please select the best fit for your research. If you are not sure, read the appropriate sections before making your selection.

☒ Life sciences ☐ Behavioural & social sciences ☐ Ecological, evolutionary & environmental sciences

For a reference copy of the document with all sections, see nature.com/authors/policies/ReportingSummary-flat.pdf

Life sciences study design

All studies must disclose on these points even when the disclosure is negative.

Sample size	The number of cells to be sampled more than 30 from 3-6 mice. We used sample size ~30 because Central Limit Theorem tells us that the sampling distribution will approximate a normal distribution.
Data exclusions	No data were excluded from the analyses
Replication	All attempts at replication were successful.
Randomization	The animals were allocated into all experimental groups were both age and sex matched.
Blinding	The investigators were not blinded for allocation because different treatments were needed (i.e. different viral injections) but were blinded for data analyses.

Reporting for specific materials, systems and methods

Materials & experimental systems

n/a	Involved in the study
☒	☐ Unique biological materials
☐	☒ Antibodies
☒	☐ Eukaryotic cell lines
☒	☐ Palaeontology
☐	☒ Animals and other organisms
☒	☐ Human research participants

Methods

n/a	Involved in the study
☒	☐ ChIP-seq
☒	☐ Flow cytometry
☒	☐ MRI-based neuroimaging

Antibodies

Antibodies used	GFP (rabbit polyclonal antibody, 1:1,000; Sigma-Aldrich, St. Louis, MO), Prox1 (mouse monoclonal, 1:500; Millipore, Burlington, MA), PCNA (goat polyclonal, 1:100; Santa Cruz Biotechnology, Dallas, TX), GFAP (rabbit polyclonal, 1:500; Dako, Agilent, Santa Clara, CA), and DCX (goat polyclonal antibody, 1:1,000; Santa Cruz Biotechnology). The sections were then incubated for 3 h with shaking at room temperature with the following secondary antibodies: Alexa 488-conjugated donkey anti-rat antibody (1:1,000; Abcam), Alexa 594-conjugated donkey anti-rat antibody (1:1,000; Abcam), Alexa 488-conjugated donkey anti-rabbit antibody (1:1,000; The Jackson Laboratory), and Alexa 488-conjugated donkey anti-goat (1:1,000; Abcam).
Validation	These antibodies have been validated by our previous studies or studies from other groups (shown on the manufacturer's websites).

Animals and other organisms

Policy information about studies involving animals; ARRIVE guidelines recommended for reporting animal research

Laboratory animals	Experiments were conducted using 6–8-week-old C57BL/6 mice of both sexes (Charles River Laboratories, Wilmington, MA) and B6.129S7-Gja1tm1Dlg/J (Cx43 floxed) mice (stock number 008039; The Jackson Laboratory, Bar Harbor, ME).
Wild animals	The study did not involve wild animals.
Field-collected samples	The study did not involve field-collected samples.
