# Supplementary material for: Lateral dispersion is required for circuit integration of newly generated dentate granule cells
Source: Nat Commun. 2019 Jul 25;10:3324. doi: 10.1038/s41467-019-11206-9 (PMC6658520; doi:10.1038/s41467-019-11206-9)
Supplement: Supplementary file 4 — Description of Additional Supplementary Information [file 41467_2019_11206_MOESM4_ESM.pdf]

Description of Additional Supplementary Files

File Name: Supplementary Movie 1
Description: *In vivo* dispersion of newly generated DGCs in freely moving adult mice.

File Name: Supplementary Movie 2
Description: Leapfrog-like dispersion of newly generated DGCs in freely moving adult mice.

File Name: Supplementary Movie 3
Description: *In vivo* dispersion of newly generated DGCs in freely moving adult mice, in which one migrating neuron exhibited an association with microvessel. Arrows point to the vessel. The neuronal migration has been tracked.

File Name: Supplementary Movie 4
Description: *In vitro* time lapse imaging of GFP+ newborn neurons at 5 days post injection.

File Name: Supplementary Movie 5
Description: *In vivo* disruption of dispersion of newly generated DGCs via knockout of connexin 43.
